# Quantifying clinical indicators for identifying and prognosticating aspiration pneumonia in Chinese geriatric patients: a retrospective cohort study integrating multivariable regression and interpretable machine learning

**DOI:** 10.3389/fmed.2026.1735311

**Published:** 2026-04-02

**Authors:** Weitao Cao, Lineng Xie, Gang Xu, Zhaoxing Lu, YuDong Hu, Zhuxiang Zhao, Qing Li, Wei Ma, Wenhui Wang, Jun Zhao

**Affiliations:** 1 Department of Respiratory and Critical Care Medicine, The Second Affiliated Hospital, School of Medicine, South China University of Technology, Guangzhou, China; 2 Department of Respiratory and Critical Care Medicine, Guangzhou First People's Hospital, Guangzhou Medical University, Guangzhou, China; 3 Department of Internal Medicine, Guangzhou Geriatric Hospital, Guangzhou, China; 4 Department of Geriatric Medicine, The Second Affiliated Hospital, School of Medicine, South China University of Technology, Guangzhou, China; 5 Department of Geriatric Medicine, Guangzhou First People's Hospital, Guangzhou Medical University, Guangzhou, China; 6 Department of Respiratory and Critical Care Medicine, The Tenth Affiliated Hospital, Southern Medical University, Dongguan People's Hospital, Dongguan, China; 7 Information and Data Centre, Guangzhou First People's Hospital, Guangzhou Medical University, Guangzhou, China; 8 Information and Data Centre, The Second Affiliated Hospital, School of Medicine, South China University of Technology, Guangzhou, China

**Keywords:** aging, aspiration pneumonia, clinical indicators, logistic regression, machine learning

## Abstract

**Objective:**

Aspiration pneumonia (AP) substantially increases mortality risk among elderly patients, necessitating prompt diagnostic recognition and prognostic stratification. However, standardized diagnostic criteria and prognostic markers remain elusive. This investigation seeks to quantify high-contribution clinical parameters for the diagnosis and evaluation of AP prognosis, elucidate nonlinear risk interactions, and establish a comprehensive risk stratification framework tailored for Chinese geriatric populations.

**Methods:**

This retrospective cohort analysis (2017–2018) enrolled 295 patients with pneumonia (aged 65–98 years) from Guangzhou First People’s Hospital. The exposure variable was the diagnosis of AP, with the primary endpoints being 3-month mortality and recurrence rates. The adjustment variables encompassed demographic characteristics, comorbidities, biomarkers, and functional status. A dual-modeling approach was implemented: (1) multivariable multinomial logistic regression; (2) interpretable random forest algorithms incorporating SHAP/PDP analyses.

**Results:**

Conventional analytical approaches demonstrated significantly elevated 3-month mortality (17.3% vs. 5.9%) and recurrence rates (42.3% vs. 17.8%) in the AP cohort (both *p* < 0.001). We identified AP as an independent predictor using multinomial logistic regression of mortality (OR = 4.082, 95%CI: 1.041–15.997) and recurrence (OR = 3.329, 95%CI: 1.370–8.090). Regarding AP prognosis, consciousness impairment, muscle strength and swallowing function emerged as pivotal assessment indicators. Machine learning insights (AUC = 0.92) identified critical quantitative thresholds associated with AP classification, including in-hospital antibiotic duration >15 days and Barthel Index ≤2, along with nonlinear interaction effects between antibiotic duration and Barthel score; antibiotic duration was interpreted as a clinical-course marker rather than a causal factor. Additionally, prognostic quantitative markers for AP were established: albumin <30 g/L, with interactive effects between albumin levels and nutritional risk.

**Conclusion:**

This dual-framework methodology amalgamates epidemiological rigor with artificial intelligence interpretability, allowing AP identification and prognostic evaluation through routine clinical parameters. This approach establishes a foundation for dynamic risk stratification in AP patients and contributes to improved clinical outcomes.

## Introduction

Pneumonia remains a leading cause of hospitalization and mortality among older adults, with a global annual incidence of 24.6–37.1 per 1,000 individuals aged ≥65 years (1). Accumulating evidence indicates that aspiration pneumonia (AP) carries higher mortality than non-aspiration pneumonia. Hospital-based and community cohorts have reported increased in-hospital and 1-year mortality risks among older adults with AP compared with community-acquired pneumonia (CAP), even after adjustment for key comorbidities (2) (3). This excess risk likely reflects the combined impact of oropharyngeal or gastric content aspiration, triggering both chemical pneumonitis and polymicrobial infection, alongside substantial comorbidity burden and a characteristic microbiological spectrum (4–6). Emerging data further suggest that AP-associated inflammation may accelerate frailty trajectories, contributing to poorer outcomes than in non-AP patients (7, 8). Given AP’s dismal prognosis and elevated mortality—particularly devastating for advanced-age patients—prompt diagnostic identification and prognostic stratification assume paramount clinical importance.

Diagnostic standardization for AP remains challenging: while guidance exists across societies, there is no universally accepted, cross-setting definition or single set of clinical criteria, and real-world implementation varies across institutions (4, 6). Moreover, dysphagia assessment differs across bedside tools and raters, introducing measurement heterogeneity that complicates consistent case ascertainment and underscores the need for objective, quantifiable indicators (5, 8). Consequently, there is a pressing need for reproducible markers that facilitate AP identification and short-term risk prediction in routine practice.

Prior studies of AP prognosis face three notable limitations. First, although elevated short-term mortality in AP versus non-AP pneumonia has been repeatedly reported, many investigations insufficiently adjust for geriatric determinants such as nutritional status, functional dependence, dysphagia, and impaired consciousness—factors likely to confound risk estimates (9, 10). Second, conventional biomarkers (e.g., C-reactive protein, procalcitonin) show heterogeneous prognostic performance across settings, with multicenter cohorts reporting substantial effect-size variability (11, 12). Third, linear models may under-represent complex risk architecture: AP arises from the interplay of aspiration burden, microbial inoculum, host frailty, and compromised airway protection. These domains likely interact in non-additive ways—threshold effects and synergistic risks are expected rather than simple linear contributions—providing *a priori* justification for methods that can reveal nonlinear interaction effects (indicating combined effects differ from the sum of individual effects) and clinically meaningful turning points.

At the same time, most existing statistical and machine-learning models in pneumonia and geriatric syndromes report modest gains in discrimination and often lack interpretability or external validation, and few are tailored to AP-specific mechanisms or quantify actionable thresholds (13, 14). Furthermore, baseline risks, comorbidity spectra, oral health, and care pathways can differ across regions and ethnicities. Therefore, quantifying AP’s independent contribution to short-term mortality and recurrence in Chinese geriatric populations is needed to support context-relevant risk stratification.

To address these gaps, we analyzed a single-center cohort of 295 hospitalized older adults with pneumonia (138 AP, 157 non-AP), integrating rigorously confounder-adjusted multivariable multinomial logistic regression with interpretable machine-learning analyses (random forest with SHAP and partial dependence). Our aims were to: (1) quantify the independent association between AP and short-term adverse outcomes in Chinese geriatric patients; (2) identify threshold effects and nonlinear interaction effects across clinical parameters; and (3) propose a pragmatic, interpretable framework for bedside risk stratification that bridges traditional epidemiology with machine learning.

## Methods

### Study population

This single-center retrospective cohort study was conducted at Guangzhou First People’s Hospital from July 1, 2017, to June 30, 2018, with follow-up ending on September 30, 2018. A total of 295 pneumonia patients aged ≥65 years were included. The exposure group comprised patients diagnosed with aspiration pneumonia, while the control group included patients with community- or hospital-acquired non-aspiration pneumonia. Aspiration pneumonia was diagnosed based on clinical criteria, radiographic evidence and swallowing function assessments (6) (detailed comprehensively in the Diagnostic Definitions section below). Exclusion criteria included age <65 years, active tuberculosis, lung malignancy, immunosuppression, severe hepatic/renal dysfunction, participation in other clinical trials within 2 months, or lack of informed consent. Data were extracted from electronic medical records (EMR) using standardized forms by two independent researchers, with discrepancies resolved by a third investigator. Demographic, laboratory, imaging, and follow-up data were systematically collected. A prespecified subsample (AP 104; NAP 101) underwent manual adjudication and conventional regression to ensure complete-case consistency and covariate harmonization; selection followed stratified random sampling by group. Main Machine Learning analyses used the full cohort (*N* = 295).

### Variables

The exposure variable, aspiration pneumonia, was defined as a binary outcome (yes/no) based on multidisciplinary consensus (respiratory physicians, radiologists, and rehabilitation specialists) within 24 h of admission, integrating clinical history, chest CT, and swallowing assessments.

### Diagnostic definitions for aspiration pneumonia

AP diagnosis required fulfillment of all three criteria:

(1) Clinical criteria: Presence of ≥2 of the following:

- Witnessed or suspected aspiration event (witnessed choking, coughing during meals, or post-meal respiratory distress reported by patient, caregivers, or nursing staff) or Known risk factors for aspiration (dysphagia, altered consciousness, neuromuscular disease, gastroesophageal reflux disease, or mechanical disruption of swallowing such as nasogastric tube placement).
- Fever (≥38.0 °C) or hypothermia (<36.0 °C).
- New or worsening cough, sputum production, or dyspnea.

(2) Radiographic criteria: Chest CT demonstrating new infiltrate(s) with characteristic distribution patterns suggestive of aspiration:

- Dependent lung zones (bilateral lower lobes, posterior segments, or right middle lobe if aspiration occurred while upright; posterior upper lobe or superior lower lobe segments if aspiration occurred while supine).
- Infiltrates were required to be new or progressive compared with prior imaging (when available) or baseline clinical status.

(3) Swallowing dysfunction criteria: Objective evidence of swallowing impairment documented by:

- Abnormal bedside swallowing assessment (Kubota Water Swallowing Test: inability to drink 30 mL water smoothly or presence of coughing, choking, or wet voice during/after swallowing).
- OR documented dysphagia based on clinical assessment (difficulty swallowing solids or liquids, prolonged meal times, avoidance of certain food textures, or requirement for dietary modifications).

Exclusion from AP diagnosis: Patients with isolated chemical pneumonitis (aspiration of gastric contents without subsequent infection, typically resolving within 48–72 h), primary bacterial pneumonia without aspiration risk factors, or infiltrates attributable to pulmonary edema, atelectasis, or other non-infectious etiologies were classified as non-AP pneumonia.

Diagnostic adjudication process: All cases underwent independent review by a respiratory physician and radiologist within 24 h of admission. Discrepancies were resolved through consensus discussion involving a third reviewer (rehabilitation specialist with dysphagia expertise). Reviewers were not blinded to clinical presentation but were instructed to apply the above criteria systematically.

We analyzed three mutually exclusive 3-month outcomes (no event, death, recurrence) using multinomial logistic regression; a composite endpoint (death or recurrence) was evaluated in prespecified sensitivity analyses. The three-level outcome (no event, recurrence, death) was analyzed using multinomial logistic regression with ‘no event’ as the reference category. This approach allows separate estimation of risk factors for recurrence and death, without assuming proportional effects across outcome levels—an appropriate choice given the potentially distinct mechanisms underlying recurrence versus mortality. Mortality was verified via hospital records or civil death registries, while recurrence required radiographic confirmation of new infiltrates and clinical diagnosis. Outcomes were recorded as binary variables (occurred/not occurred) and adjudicated by blinded assessors. Covariates included age (continuous), sex (binary), nutritional risk (NRS-2002 score ≥3), albumin (serum levels within 24 h, continuous), cerebral infarction (radiologically confirmed), dysphagia (clinical assessment + Kubota Water Swallowing Test), C-reactive protein, white blood cell count, urea nitrogen (measured within 24 h), impaired consciousness (defined by documented decreased alertness and/or Glasgow Coma Scale ≤12 at assessment), and Barthel Index (recorded on a 0–100 scale and normalized to 0–4 for Machine Learning analyses), and other relevant variables. These variables were selected based on established associations with aspiration pneumonia outcomes in prior literature. For conventional regression, missing data (<5%) were handled using multiple imputation by chained equations (MICE).

Variable Selection: Variables were selected based on prior AP literature and clinical relevance. For regression models, we used stepwise selection (entry *p* < 0.05, retention *p* < 0.10). For machine learning, we used all candidate variables without preselection to capture complex patterns.

### Traditional data analysis

Patients with pneumonia (*N* = 295) were stratified into aspiration pneumonia (AP, *n* = 138) and non-aspiration pneumonia (NAP, *n* = 157) groups based on multidisciplinary diagnostic criteria incorporating clinical history, chest CT imaging, and swallowing function evaluations. A randomly sampled subset (AP: *n* = 104; NAP: *n* = 101) underwent conventional statistical analysis using multivariable regression models.

Descriptive Statistics: Continuous variables were expressed as mean ± standard deviation (normally distributed), while categorical variables were described as frequency and percentage. Group differences were assessed using χ^2^ tests (categorical variables), Student’s *t*-tests (normally distributed continuous variables), or Mann–Whitney U tests (non-normally distributed continuous variables).

Comparative Analyses: Multinomial Logistic Regression model.

### Machine learning analysis

This retrospective study included a total of 295 patients with pneumonia. Patient diagnosis and group allocation were strictly conducted according to the clinical criteria detailed in the preceding sections. The entire cohort (*N* = 295) was utilized in the machine learning-based analyses to ensure the comprehensive evaluation of clinical data. Missing data (4.8%) were imputed using MissForest, a random forest-based imputation method that has demonstrated superiority over traditional techniques in clinical datasets (15). Continuous variables were standardized using z-scores, and the dataset was partitioned into training (70%) and testing (30%) sets. To address class imbalance, we applied the SMOTE-ENN hybrid resampling technique, which balances sensitivity and specificity more effectively than SMOTE alone in pneumonia research (16, 17). The Random Forest classifier (scikit-learn v1.60) was optimized via Bayesian optimization with 100 iterations, selecting 500 trees (n_estimators) and max_depth = 8 through 5-fold cross-validation (18). Feature randomization used log2(p) features per split (*p* = 25). Model performance was evaluated on the held-out test set using AUC-ROC, sensitivity, specificity, and accuracy. Given the limited size of the held-out prognostic test set, we focused on discrimination metrics in this study, calibration assessment is planned for future external validation. For prognosis prediction within AP patients, we used an optimized random forest classifier to predict 3-month mortality. Top predictors were clinically contextualized using SHAP (SHapley Additive exPlanations, a method quantifying each predictor’s contribution to model predictions) values (SHAP v0.42), which overcome Gini importance’s bias toward high-cardinality features (19). Throughout the machine learning workflows, MissForest imputation and SMOTE-ENN resampling were strictly applied only within the training folds during nested cross-validation; the held-out test set remained completely untouched for the final evaluation. Furthermore, sensitivity analyses were conducted by comparing models without resampling (using class weights), with SMOTE only, and with ENN only.

### Ethic statement

This investigation received ethical clearance from the Institutional Review Board of Guangzhou First People’s Hospital (approval number: K-2017-094-02).

## Results

### Baseline characteristics and clinical outcomes

Comparative analysis of 104 AP patients versus 101 NAP patients revealed substantial clinical and prognostic disparities. The AP cohort demonstrated significantly advanced age (87.99 ± 5.67 vs. 84.42 ± 8.52 years, *p* < 0.001), while gender distribution remained comparable (male proportion: 77.9% vs. 82.2%, *p* = 0.442). Laboratory parameters indicated elevated B-type natriuretic peptide levels (2948.32 ± 4427.71 vs. 1897.85 ± 2538.50, *p* = 0.039) and leukocyte counts (11.41 ± 4.48 vs. 9.93 ± 4.74 × 10^9^/L, *p* = 0.023) in the AP group, accompanied by prolonged antibiotic duration (15.22 ± 7.34 vs. 11.97 ± 4.82 days, *p* < 0.001). Conversely, cholesterol levels (3.26 ± 1.05 vs. 3.61 ± 1.00 mmol/L, *p* = 0.014) and albumin concentrations (30.62 ± 4.34 vs. 33.31 ± 4.32 g/L, *p* < 0.001) were significantly lower in the AP group compared to the NAP group.

Comorbidity profiles differed markedly: AP patients exhibited higher stroke prevalence (86.5% vs. 54.5%, *p* < 0.001), whereas COPD was more prevalent in NAP patients (49.5% vs. 32.7%, *p* = 0.014). Clinical manifestations significantly associated with AP included consciousness impairment (11.5% vs. 2.0%, *p* = 0.007), muscle weakness (80.8% vs. 51.5%, *p* < 0.001), and nutritional risk (46.2% vs. 20.8%, *p* < 0.001). Three-month follow-up demonstrated substantially elevated mortality (17.3% vs. 5.9%) and pneumonia recurrence rates (42.3% vs. 17.8%, *p* < 0.001) in the aspiration pneumonia cohort, underscoring its association with adverse outcomes (Table 1).

**Table 1:** Clinical characteristics and laboratory parameters of patients with and without aspiration pneumonia.

Characteristics	Aspiration pneumonia (*n* = 104)	Non-aspiration pneumonia (*n* = 101)	*P*-value
Demographics			
Age (years)	87.99 ± 5.67	84.42 ± 8.52	<0.001^***^
Male sex, *n* (%)	81 (77.9)	83 (82.2)	0.442
Laboratory parameters			
BNP (pg/mL)	2948.32 ± 4427.71	1897.85 ± 2538.50	0.039^*^
CRP (mg/L)	57.19 ± 45.98	54.89 ± 57.03	0.751
PCT (ng/mL)	1.42 ± 4.31	0.92 ± 3.22	0.356
Cholesterol (mmol/L)	3.26 ± 1.05	3.61 ± 1.00	0.014^*^
WBC (×10^9^/L)	11.41 ± 4.48	9.93 ± 4.74	0.023^*^
Neutrophils (%)	79.83 ± 12.94	76.71 ± 12.09	0.076
Platelets (×10^9^/L)	252.14 ± 96.15	222.49 ± 84.59	0.020^*^
Hemoglobin (g/L)	11.55 ± 8.92	11.67 ± 7.99	0.916
BUN (mmol/L)	8.64 ± 6.16	8.14 ± 4.69	0.515
Scr (μmol/L)	90.82 ± 74.51	101.65 ± 52.03	0.230
Albumin (g/L)	30.62 ± 4.34	33.31 ± 4.32	<0.001^***^
Comorbidities, *n* (%)			
COPD	34 (32.7)	50 (49.5)	0.014^*†^
Asthma	2 (1.9)	4 (4.0)	0.387^†^
Bronchiectasis	10 (9.6)	11 (10.9)	0.763^†^
Lung cancer	3 (2.9)	4 (4.0)	0.672^†^
Hypertension	73 (70.2)	69 (68.3)	0.771^†^
Stroke	90 (86.5)	55 (54.5)	<0.001^***†^
Dementia	47 (45.2)	10 (9.9)	<0.001^***†^
Parkinson’s disease	14 (13.5)	5 (5.0)	0.022^*†^
Diabetes	32 (30.8)	34 (33.7)	0.657^†^
Clinical features, *n* (%)			
Dysphagia	13 (12.5)	5 (5.0)	0.056^†^
Consciousness disorder	12 (11.5)	2 (2.0)	0.007^**†^
Decreased muscle strength	84 (80.8)	52 (51.5)	<0.001^***†^
Nutritional risk	48 (46.2)	21 (20.8)	<0.001^***†^
Antibiotic duration (days)	15.22 ± 7.34	11.97 ± 4.82	<0.001^***^
3-month outcomes, *n* (%)			<0.001^***†^
No adverse events	42 (40.4)	77 (76.2)	
Death	18 (17.3)	6 (5.9)	
Recurrent pneumonia	44 (42.3)	18 (17.8)	

Continuous variables are presented as mean ± standard deviation; categorical variables are presented as *n* (%). ^*^P < 0.05, ^**^P < 0.01, ^***^P < 0.001 ^†^*P*-values calculated using Chi-square test or Fisher’s exact test for categorical variables; all other *P*-values calculated using Student’s *t*-test.

BNP, B-type natriuretic peptide; CRP, C-reactive protein; PCT, procalcitonin; WBC, white blood cell count; BUN, blood urea nitrogen; Scr, serum creatinine; COPD, chronic obstructive pulmonary disease.

### Multivariate risk factor analysis

Based on statistically significant variables from Table 1 and clinically relevant pneumonia determinants, we conducted multinomial logistic regression analysis incorporating 16 clinical parameters. Our analysis identified AP as an independent predictor of 3-month mortality risk escalation (vs. NAP: OR = 4.082, *p* = 0.044), conferring a 4.082-fold increased odds compared to non-aspiration cases. Additionally, AP elevated 3-month recurrence risk by 3.329-fold (vs. NAP: OR = 3.329, *p* = 0.008).

Regarding mortality risk, four additional parameters achieved statistical significance: advanced age (OR = 1.199, 95% CI: 1.047–1.372, *p* = 0.009), consciousness impairment (OR = 43.50, 95% CI: 3.0–650.0, *p* = 0.006), nutritional risk (OR = 3.792, 95% CI: 1.11–12.82, *p* = 0.023) and BUN (OR = 1.149, 95% CI: 1.034–1.278, *p* = 0.010). Consciousness impairment demonstrated the strongest association, amplifying mortality risk nearly 43.5-fold. For pneumonia recurrence, two parameters showed statistical significance: advanced age (OR = 1.092, 95% CI: 1.010–1.180, *p* = 0.027), consciousness impairment (OR = 114.085, 95% CI: 1.221–166.667, *p* = 0.034) (Table 2).

**Table 2:** Multinomial logistic regression analysis of risk factors for mortality and pneumonia recurrence during 3-month follow-up.

Variables	Death		Recurrent pneumonia	
	OR (95% CI)	*P* value	OR (95% CI)	*P* value
Demographic and clinical characteristics				
Age	1.199 (1.047–1.372)	0.009^**^	1.092 (1.010–1.180)	0.027^*^
Gender (Male vs. female)	2.785 (0.503–15.434)	0.241	1.583 (0.594–4.216)	0.358
Consciousness disorder (No vs. Yes)	0.023 (0.002–0.331)	0.006^**^	0.071 (0.006–0.819)	0.034^*^
Aspiration pneumonia (Yes vs. No)	4.082 (1.041–15.997)	0.044^*^	3.329 (1.370–8.090)	0.008^**^
Dysphagia (No vs. Yes)	0.196 (0.030–1.292)	0.090	0.900 (0.205–3.953)	0.889
Stroke (No vs. Yes)	1.082 (0.203–5.760)	0.926	2.207 (0.840–5.797)	0.108
Nutritional risk (No vs. Yes)	0.264 (0.078–0.897)	0.033^*^	0.722 (0.327–1.594)	0.420
Muscle strength (Normal vs. Decreased)	0.859 (0.180–4.103)	0.849	1.504 (0.640–3.533)	0.349
Dementia (No vs. Yes)	2.204 (0.561–8.660)	0.258	1.197 (0.508–2.823)	0.681
COPD (No vs. Yes)	0.310 (0.089–1.078)	0.065	0.599 (0.280–1.284)	0.188
Parkinson’s disease (No vs. Yes)	2.943 (0.157–55.250)	0.471	0.755 (0.224–2.547)	0.650
Laboratory and treatment parameters				
WBC	1.095 (0.973–1.234)	0.132	1.024 (0.938–1.118)	0.599
PCT	0.789 (0.589–1.056)	0.111	0.880 (0.734–1.055)	0.167
Albumin	0.968 (0.845–1.109)	0.641	1.042 (0.952–1.142)	0.371
BUN	1.149 (1.034–1.278)	0.010^*^	1.034 (0.954–1.121)	0.420
Antibiotic use duration	1.011 (0.926–1.105)	0.803	1.019 (0.960–1.081)	0.538

OR, odds ratio; CI, confidence interval; PCT, procalcitonin; WBC, white blood cell count; BUN, blood urea nitrogen; COPD, chronic obstructive pulmonary disease. ^*^Statistically significant (*P* < 0.05).

Reference category was “No adverse events”. Variables were entered using stepwise selection (entry *p* < 0.05, removal *p* < 0.10).

These findings underscore aspiration pneumonia’s elevated mortality and recurrence risks compared to non-aspiration variants, emphasizing the critical importance of early identification. The statistical analysis revealed that advanced age, consciousness impairment, and nutritional risk constitute high-risk mortality factors while simultaneously serving as important aspiration pneumonia indicators. Consequently, quantifying clinical parameters plays a pivotal role in aspiration pneumonia recognition and clinical guidance, prompting our machine learning analysis of AP versus NAP patient cohorts to identify quantitative biomarkers.

### Machine learning model for AP identification

**Figure 1:**
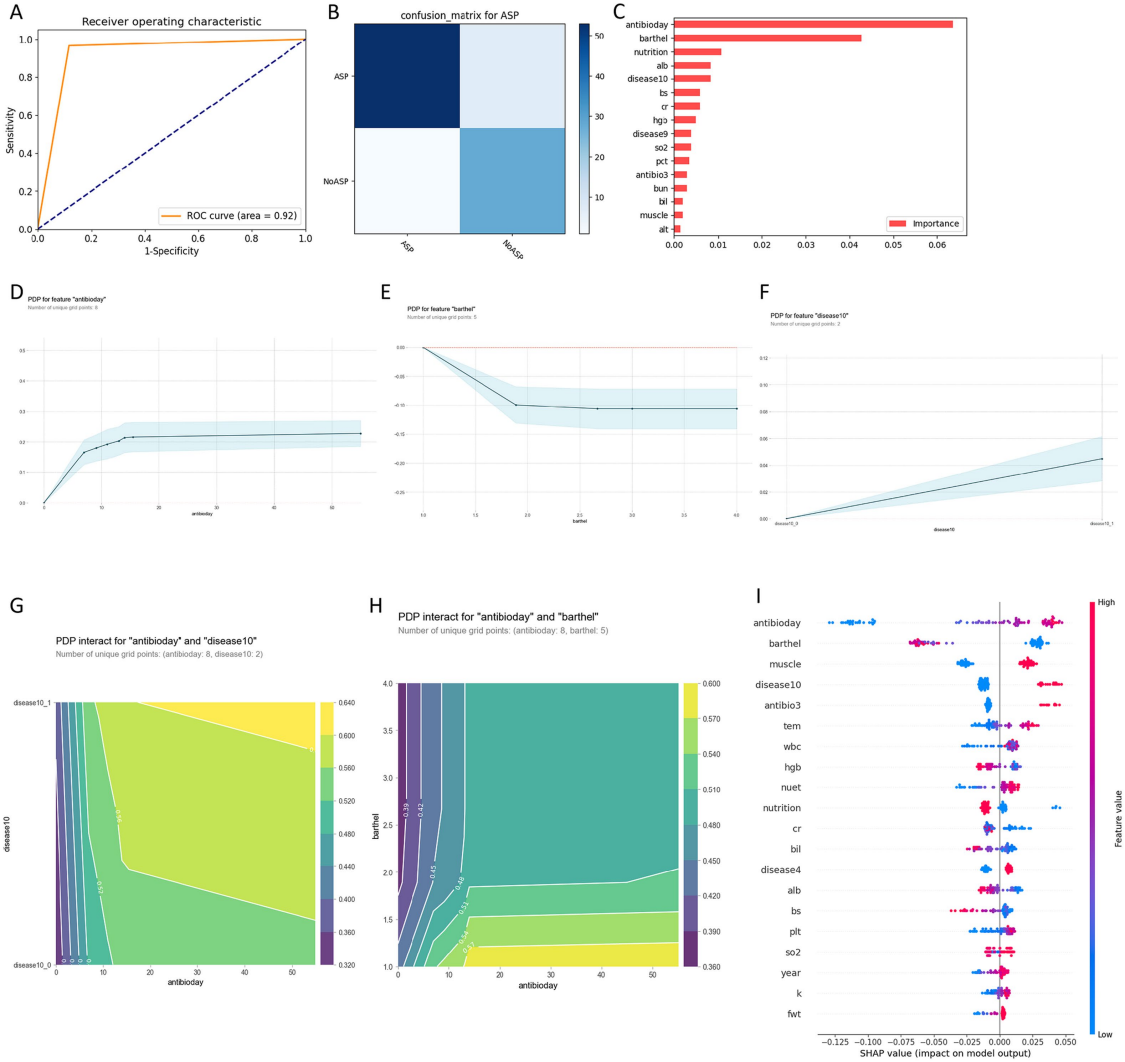
Machine learning model performance and feature analysis for aspiration pneumonia (AP) versus non-aspiration pneumonia (NAP) classification. **(A)** Receiver operating characteristic (ROC) curve demonstrating model performance with area under the curve (AUC) = 0.92. **(B)** Confusion matrix showing classification accuracy for aspiration pneumonia prediction. **(C)** Feature importance ranking based on model contribution, with antibiotic days, Barthel index, and nutrition status as top predictors. **(D–F)** Partial dependence plots (PDPs) illustrating individual feature effects on prediction probability. **(G)** PDP interaction plot between antibiotic days and dementia status (disease10). **(H)** PDP interaction plot between antibiotic days and Barthel index. **(I)** SHAP (SHapley Additive exPlanations) value plot displaying feature contributions to individual predictions. Features are ranked by importance (top to bottom), with each dot representing a single prediction. Colors indicate feature values (red = high, blue = low), and the x-axis shows the impact on model output (positive values increase prediction, negative values decrease prediction). Abbreviations: Antibody, HP antibody (*Helicobacter pylori* antibody); Barthel, Barthel Index; muscle, muscle strength; disease10, dementia; antibio3, imipenem; tem, temperature; WBC, white blood cell count; HGB, hemoglobin; nuet, neutrophil percentage; nutrition, nutrition risk; cr, creatinine; bil, bilirubin; disease4, cerebral infarction; alb, albumin; bs, blood sugar; plt, platelet count; so2, oxygen saturation; year, age; k, potassium; fwt, frog water test.

The identification model demonstrated exceptional discriminatory performance: area under the curve (AUC) reached 0.92 (95% confidence interval, 0.87–0.95) with overall accuracy of 92.01% (Figure 1A). The confusion matrix revealed robust diagnostic capability (28 true positives, 53 true negatives, 7 false positives, 1 false negative), achieving 97% sensitivity and 88% specificity (Figure 1B). Feature importance analysis identified critical indicators, with antibiotic duration, Barthel Index, nutritional status, albumin levels, and dementia emerging as the most significant predictors (Figure 1C). Partial dependence plot (PDP, visualizing the marginal effect of a predictor on outcome probability) analysis revealed a distinct 15-day threshold effect for antibiotic duration: AP prediction contribution values rapidly ascended from 0 to 0.20 during the 0–10 day period, reached a critical inflection point at 10–15 days, then plateaued beyond 15 days (0.22–0.23) (Figure 1D). The Barthel Index exhibited a pronounced threshold effect at 2.0: patients with severe functional impairment (Barthel = 1.0) demonstrated the highest aspiration pneumonia prediction contribution (approaching 0), with a critical threshold transition at Barthel = 2.0 (Moderately impaired patients), where prediction values significantly declined and stabilized at −0.11 (Figure 1E). Dementia manifested a clear binary effect, increasing AP prediction contribution by approximately 0.04 compared to non-dementia patients (Figure 1F).

Furthermore, we analyzed quantitative factor interactions. Antibiotic duration and dementia exhibited significant interaction effects. Among non-dementia patients, prediction probability gradually increased from 0.32 to 0.52 over 0–50 days of antibiotic use; dementia patients showed dramatic AP prediction contribution escalation after 10 days of antibiotic use, with prediction probability reaching 0.64 during prolonged treatment, demonstrating nonlinear synergistic enhancement patterns (Figure 1G).

Antibiotic duration and Barthel Index demonstrated significant interaction effects, presenting functional status-dependent stratified risk patterns. Severely impaired patients (Barthel = 1.0) showed prediction probabilities of approximately 0.36 during 0–10 days of antibiotic use, with dramatic risk escalation occurring at the 10–20 day interval, reaching 0.60 during prolonged use (>30 days). Moderately impaired patients (Barthel = 2.0) displayed relatively moderate risk progression, with prediction probability increasing from approximately 0.42 to 0.52. Patients with mild impairment or normal function (Barthel = 3.0–4.0) demonstrated lowest sensitivity to antibiotic duration, maintaining gradual risk progression throughout the 0–50 day range, with prediction probabilities sustained at 0.45–0.50 levels (Figure 1H). SHAP analysis confirmed antibiotic duration, Barthel Index, muscle condition, and dementia as the most influential predictive factors, with antibiotic duration exerting the strongest overall model prediction impact, validating the stratified importance and interaction patterns observed in partial dependence plot analysis (Figure 1I).

### Prognostic analysis within AP patients

Having established quantitative indicators for AP identification, we next investigated which clinical parameters best predicted adverse outcomes specifically within the AP patient cohort. Initially, we employed conventional statistical methods for analysis. Based on three outcomes during 3-month follow-up (no adverse event, death, pneumonia recurrence), we stratified 104 elderly aspiration pneumonia patients into three groups, comparing demographic data and related clinical examination results.

The mortality group exhibited the highest B-type natriuretic peptide levels (5707.46 ± 7535.52 pg./mL, *p* = 0.005), significantly exceeding both the event-free group (1737.06 ± 2334.85) and recurrence group (2975.78 ± 3833.01), suggesting cardiac dysfunction may serve as a mortality outcome predictor. Among the different outcome groups, the cholesterol level was lowest in the death group (2.50 ± 0.79, *p* = 0.023), while there was no significant difference in cholesterol levels between the no adverse events group (3.43 ± 1.10) and the recurrence group (3.40 ± 0.97) (Table 3).

**Table 3:** Comprehensive clinical characteristics and outcomes of elderly patients with aspiration pneumonia at baseline and 3-month follow-up (*N* = 104).

Characteristic	No adverse event (*n* = 42)	Death (*n* = 18)	Recurrent pneumonia (*n* = 44)	*P*-value
Laboratory parameters				
BNP (pg/mL)	1737.06 ± 2334.85	5707.46 ± 7535.52	2975.78 ± 3833.01	0.005^**^
CRP (mg/L)	60.79 ± 50.65	79.11 ± 50.26	44.78 ± 35.31	0.003^**^
PCT (ng/mL)	2.16 ± 6.44	1.16 ± 1.62	0.81 ± 1.73	0.336
Cholesterol (mmol/L)	3.43 ± 1.10	2.50 ± 0.79	3.40 ± 0.97	0.023^*^
BUN (mmol/L)	7.17 ± 4.32	12.21 ± 9.41	8.58 ± 5.54	0.013^*^
Scr (μmol/L)	82.58 ± 56.58	94.33 ± 32.70	97.26 ± 98.54	0.647
WBC (×10^9^/L)	11.32 ± 4.62	12.35 ± 6.43	11.10 ± 3.29	0.604
Neutrophils (%)	78.78 ± 15.38	82.73 ± 7.67	79.64 ± 12.15	0.556
Platelets (×10^9^/L)	244.81 ± 85.71	273.78 ± 147.04	250.30 ± 79.61	0.561
Hemoglobin (g/L)	108.48 ± 22.81	97.06 ± 17.66	100.64 ± 17.13	0.069
Albumin (g/L)	31.07 ± 4.60	28.53 ± 4.69	31.05 ± 3.76	0.078
Demographics				
Age (years)	86.02 ± 5.99	90.94 ± 6.16	88.66 ± 4.47	0.004^*^
Male sex, *n* (%)	33 (78.6)	15 (83.3)	33 (75.0)	0.766^†^
Comorbidities, *n* (%)				
COPD	13 (31.0)	8 (44.4)	13 (29.5)	0.500^†^
Asthma	1 (2.4)	0 (0)	1 (2.3)	0.807^†^
Bronchiectasis	6 (14.3)	1 (5.6)	3 (6.8)	0.408^†^
Lung cancer	0 (0)	1 (5.6)	2 (4.5)	0.343^†^
Stroke	32 (76.2)	17 (94.4)	41 (93.2)	0.039^*†^
Dementia	18 (42.9)	8 (44.4)	21 (47.7)	0.900^†^
Parkinson’s disease	7 (16.7)	1 (5.6)	6 (13.6)	0.512^†^
Hypertension	30 (71.4)	11 (61.1)	32 (72.7)	0.646^†^
Diabetes	16 (38.1)	3 (16.7)	13 (29.5)	0.250^†^
Clinical features, *n* (%)				
Consciousness disorder	1 (2.4)	5 (27.8)	6 (13.6)	0.016^*†^
Dysphagia	3 (7.1)	5 (27.8)	5 (11.4)	0.082^†^
Decreased muscle strength	33 (78.6)	14 (77.8)	37 (84.1)	0.761^†^
Nutritional risk	17 (40.5)	12 (66.7)	19 (43.2)	0.91^†^
Antibiotic Duration (days)	15.55 ± 7.10	15.33 ± 7.15	14.86 ± 7.79	0.744

Data are presented as mean ± SD unless otherwise specified. *P* < 0.05, *P* < 0.01, *P* < 0.001. ^†^*P*-values calculated using χ^2^ test or Fisher’s exact test; all other *P*-values calculated using ANOVA.

BNP, B-type natriuretic peptide; CRP, C-reactive protein; PCT, procalcitonin; WBC, white blood cell count; BUN, blood urea nitrogen; Scr, serum creatinine; COPD, chronic obstructive pulmonary disease. ^*^P < 0.05, ^**^P < 0.01.

Advanced age correlated strongly with mortality risk, with the death group showing significantly higher age (90.94 ± 6.16) than the no adverse event group (86.02 ± 5.99) and recurrence group (88.66 ± 4.47, *p* = 0.004). Consciousness impairment occurred most frequently in the mortality group (27.8% vs. no adverse event group 2.4% and recurrence group 13.6%, *p* = 0.016), while dysphagia showed a non-significant elevation trend in the mortality group (27.8% vs. 7.1%, *p* = 0.082). Inflammatory markers (CRP, *p* = 0.063; procalcitonin, *p* = 0.336), comorbidities (COPD, diabetes), and antibiotic duration showed no significant inter-group differences (all *p* > 0.05) (Table 3).

We selected 13 clinical parameters for multinomial logistic regression analysis. Advanced age presented differential risk associations: each additional year increased mortality risk by 34.7% (OR = 1.347, 95% CI = 1.132–1.602, *p* = 0.001) and recurrence risk by 13.9% (OR = 1.139, 95% CI = 1.016–1.277, *p* = 0.026). Consciousness impairment substantially elevated mortality risk (OR = 40.000, 95% CI = 1.942–824.000, *p* = 0.020), while normal swallowing function (OR = 0.069 vs. dysphagia, 95% CI = 0.005–0.927, *p* = 0.044) and muscle strength (OR = 0.108 vs. muscle weakness, 95% CI = 0.014–0.843, *p* = 0.034) emerged as protective factors reducing mortality risk. Regarding pneumonia recurrence, advanced age was the only clinical factor showing statistical significance, while all other clinical indicators demonstrated no significant differences (all *p* > 0.05) (Table 4).

**Table 4:** Comprehensive multinomial logistic regression analysis of risk factors for adverse outcomes in elderly patients with aspiration pneumonia.

Variable	Death		Recurrent pneumonia	
	OR (95% CI)	*P* value	OR (95% CI)	*P* value
Demographic and clinical characteristics				
Age	1.364 (1.126–1.653)	0.002^**^	1.124 (1.005–1.257)	0.040^*^
Gender (Male)	1.495 (0.222–10.065)	0.680	0.816 (0.251–2.653)	0.735
Consciousness disorder (Yes vs. No)	40.000 (1.942–824.000)	0.017^*^	7.143 (0.522–97.778)	0.141
Dysphagia (No vs. Yes)	0.069 (0.005–0.927)	0.044^*^	0.417 (0.058–2.987)	0.384
Stroke (No vs. Yes)	12.499 (0.315–496.546)	0.179	3.930 (0.869–17.770)	0.075
Nutritional risk (No vs. Yes)	0.282 (0.053–1.506)	0.139	1.044 (0.381–2.863)	0.933
Muscle strength (Normal vs. Decreased)	0.108 (0.014–0.843)	0.034^*^	0.705 (0.198–2.511)	0.589
COPD (No vs. Yes)	0.190 (0.034–1.077)	0.061	0.831 (0.292–2.363)	0.728
Dementia (No vs. Yes)	3.493 (0.649–18.813)	0.145	1.414 (0.518–3.859)	0.499
Laboratory parameters				
WBC (×10^9^/L)	1.082 (0.932–1.256)	0.299	1.013 (0.894–1.148)	0.839
BUN (mmol/L)	1.119 (0.978–1.281)	0.101	1.060 (0.952–1.181)	0.285
PCT (ng/mL)	0.702 (0.478–1.032)	0.072	0.852 (0.678–1.069)	0.167
Treatment factors				
Antibiotic duration (days)	0.994 (0.903–1.093)	0.894	0.991 (0.929–1.057)	0.781

Data are presented as OR (95% CI). OR, odds ratio; CI, confidence interval. ^*^P < 0.05, ^**^P < 0.01. WBC, white blood cell count; BUN, blood urea nitrogen; PCT, procalcitonin; COPD, chronic obstructive pulmonary disease.

Reference category: no adverse event group.

### Machine learning model for prognosis prediction

**Figure 2:**
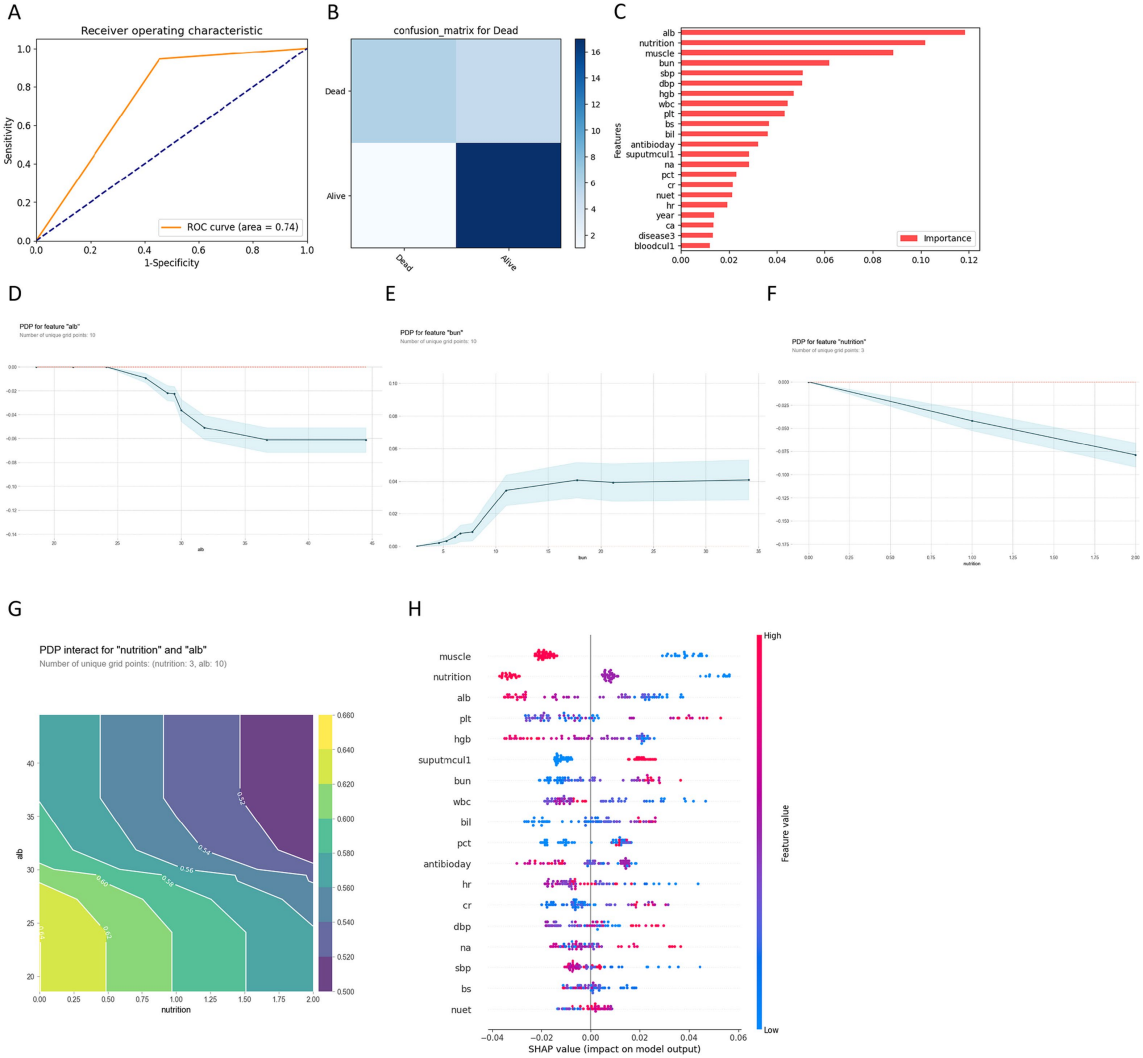
Machine learning model development and feature analysis for mortality prediction in aspiration pneumonia patients. **(A)** Receiver operating characteristic (ROC) curve demonstrating good model discrimination for mortality prediction with area under the curve (AUC) = 0.74. **(B)** Confusion matrix showing classification performance for mortality (Dead) versus survival (Alive) prediction in aspiration pneumonia patients. **(C)** Feature importance ranking identifying albumin (Alb), muscle mass, Barthel index (Barthel), and other clinical parameters as key predictors of mortality. **(D–F)** Partial dependence plots (PDPs) illustrating individual feature effects on mortality probability. **(G)** Two-dimensional PDP interaction between nutrition status and albumin levels. **(H)** SHAP (SHapley Additive exPlanations) summary plot displaying feature contributions to individual mortality predictions. Features are ranked by importance (top to bottom), with each dot representing a single prediction. Point colors represent feature values (red = high, blue = low), and the x-axis shows the impact on model output (positive values increase mortality risk, negative values decrease mortality risk). Abbreviations: muscle, muscle strength; nutrition, nutrition risk; Alb, albumin; plt, platelet count; Hgb, hemoglobin; suputmcul1, positive sputum culture; BUN, blood urea nitrogen; WBC, white blood cell count; Bil, bilirubin; PCT, procalcitonin; antibioday, antibiotic duration days; hr., heart rate; cr, creatinine; DBP, diastolic blood pressure; na, sodium; SBP, systolic blood pressure; bs, blood sugar; nuet, neutrophil percentage.

To complement traditional regression findings and identify nonlinear prognostic thresholds, we applied machine learning analysis to the AP cohort. Concurrently, we employed optimized random forest algorithms for machine learning analysis within the aspiration pneumonia population to identify quantitative indicators affecting mortality prognosis (Figure 2). Training and testing set accuracies reached 95.38 and 79.31% respectively, with AUC of 0.74 (Figure 2A). Given the small held-out test sample (*n* = 29), calibration and performance estimates should be interpreted cautiously. The confusion matrix based on 29 samples revealed 17 true positives and 6 true negatives (Figure 2B). Feature importance analysis indicated that albumin, nutritional status, muscle strength, and blood urea nitrogen constituted the top four predictors of 3-month mortality risk, dominating model predictive capability (Figure 2C).

Partial dependence plot analysis demonstrated a distinct albumin threshold effect with a critical value of 30 g/L. Below this threshold, mortality risk escalated dramatically as albumin declined, while risk reduction plateaued above 30 g/L (−0.06) (Figure 2D). BUN exhibited a clear threshold effect with a critical value of 10 mmol/L. Below this threshold, mortality risk rapidly increased with BUN elevation, while risk increment plateaued above 10 mmol/L (0.04) (Figure 2E). Nutritional status demonstrated linear protective effects on 3-month mortality in elderly aspiration pneumonia patients. Compared to severe nutritional risk (0 points), moderate risk (1 point) reduced mortality risk by 0.05, while no nutritional risk (2 points) further decreased risk to −0.08, presenting consistent dose–response benefits without threshold effects (Figure 2F).

Albumin, as the indicator with highest model prediction contribution, exhibited interaction effects with nutritional risk. The partial dependence plot for nutritional risk-albumin interaction revealed significant interaction effects on 3-month mortality risk in elderly aspiration pneumonia patients. Peak mortality risk (0.62–0.66) occurred in patients with severe nutritional risk (0 points) combined with low albumin levels (<25 g/L). Conversely, lowest risk (0.50–0.54) appeared in patients with no nutritional risk (2 points) and high albumin levels (>35 g/L) (Figure 2G). SHAP analysis confirmed albumin, nutritional risk, and urea nitrogen as important predictive factors, with albumin and nutritional risk exerting the strongest overall model prediction influence, validating the stratified importance and interaction effects observed in partial dependence plot analysis (Figure 2H).

## Discussion

### Summary of main findings

This retrospective cohort investigation enrolled 295 elderly patients. We integrated conventional statistical analysis with interpretable machine learning (SHAP/random forest) to establish quantitative indicators. This dual-framework approach combines hypothesis-driven analysis with data-driven pattern recognition for AP identification and prognosis. This approach addresses limitations of traditional linear models by capturing potential nonlinear interactions such as nutrition-inflammation imbalances. We found that aspiration pneumonia independently elevated mortality risk by 4.082-fold and recurrence risk by 3.329-fold, with consciousness impairment demonstrating a substantial mortality association (OR = 43.50, 95% CI: 3.0–650.0).

### Comparison with previous studies

Our findings demonstrate both convergence and divergence with previous studies on AP risk stratification. Consistent with Komiya et al. in their meta-analysis (20), we observed significantly higher 3-month mortality risk in AP versus non-AP patients (HR = 1.8 vs. 1.32–2.01), with consciousness disorder (20), age (20), dysphagia (21) and COPD complication (21) emerging as core prognostic determinants. This aligns with previous studies. Methodologically, our study achieved more precise quantification of AP’s independent risk contribution through incorporation of 12 clinically relevant covariates, surpassing certain investigations (22). Addressing Wynants’ critique regarding traditional logistic regression limitations in capturing nonlinear relationships (23), our PDP and SHAP analysis revealed quantitative thresholds of albumin levels (critical value 30/L), complementing Shimizu A, et al.’s inflammation-nutrition imbalance research (22, 24). Our differentiation from Wang et al. (*Chinese Journal of Geriatrics* 2022) manifests through random forest algorithms demonstrating superior feature selection capabilities and developing more practical screening tools for primary care settings. These approaches may contribute to risk stratification in resource-constrained settings, though external validation is needed. Beyond methodological innovations, our quantitative thresholds have direct clinical implications.

### Clinical implications of quantitative thresholds

By integrating interpretable machine learning with traditional statistical modeling, we established quantitative indicators for AP identification in older Chinese populations. Notably, our model identified a 15-day antibiotic duration threshold that effectively distinguished AP from NAP patients. However, this finding should be interpreted strictly as a risk marker rather than a causal factor. Prolonged antibiotic use likely reflects underlying disease severity, delayed clinical resolution, and overall treatment complexity. Patients requiring more than 15 days of antibiotic therapy frequently present with more severe infections or a greater comorbidity burden. Consequently, this threshold serves to identify high-risk phenotypes rather than to advocate for arbitrary restrictions on antibiotic duration.

Our findings provide inaugural quantitative evidence for specific temporal thresholds where dysbiotic effects achieve diagnostic discriminatory significance for aspiration pneumonia.

The Barthel Index threshold effect at 2.0 reflects nonlinear relationships between functional status and aspiration risk. Severely impaired patients (Barthel < 2.0) constitute high-risk phenotype aspiration pneumonia populations. This is due to severely compromised neuromuscular control and diminished pharyngolaryngeal protective reflexes. Patients with moderate or superior functional status (Barthel ≥ 2.0) maintain relatively stable and lower aspiration risks (6, 9). This 2.0 threshold provides clinicians with explicit risk stratification criteria, facilitating targeted preventive measures and resource allocation for severely functionally impaired patients.

Dementia emerges as a crucial AP risk assessment indicator: dementia patients experience diminished swallowing awareness and delayed pharyngolaryngeal protective reflexes due to cognitive impairment, linearly escalating aspiration pneumonia risk (25). In clinical assessments, comorbid dementia should serve as an essential foundation for aspiration pneumonia early warning and intervention management.

Our analysis revealed an interaction between antibiotic duration and dementia: Dementia patients showed higher AP risk when antibiotic duration exceeded 10 days. This likely reflects greater vulnerability of cognitively impaired patients with both baseline dysphagia and severe illnesses requiring prolonged treatment. This finding suggests enhanced aspiration precautions (swallowing assessments, dietary modifications) in dementia patients requiring extended therapy, rather than restricting antibiotic duration.

The Barthel Index-antibiotic duration interaction effect reflects time-dependent functional status modulation of infection risk. Severely functionally impaired patients demonstrate heightened sensitivity to prolonged antibiotic usage due to severely compromised swallowing function, immunosuppression, and bed-related complications, particularly showing significant risk escalation after 10–20 days of usage. This stratified effect suggests individualized antibiotic strategies based on patient functional status, with severely impaired patients requiring enhanced aspiration pneumonia vigilance during prolonged antibiotic therapy. To our knowledge, evidence quantifying actionable quantitative thresholds and interactions in AP has been limited. Our findings suggest clinically useful patterns, while requiring external validation before widespread adoption.

Subsequently, we identified aspiration pneumonia (AP) prognostic indicators through conventional statistical analysis and machine learning models. Multinomial logistic regression analysis indicated consciousness impairment and age as primary predictive factors. Machine learning analysis prioritized albumin levels, nutritional status, and urea nitrogen as principal predictive features, demonstrating complementary prognostic information by identifying clinically interpretable risk markers and thresholds.

The 30 g/L albumin threshold provides explicit clinical guidance for risk stratification in elderly aspiration pneumonia patients. This threshold effect suggests maintaining albumin levels above 30 g/L may support risk stratification. Patients below this level face significantly elevated mortality risk (26). They may benefit from aggressive nutritional intervention and albumin supplementation. Whether nutritional support or albumin administration improves outcomes requires prospective validation.

The 10 mmol/L BUN threshold reflects renal impairment and fluid balance disruption in elderly aspiration pneumonia patients (27). BUN elevation beyond this threshold indicates compromised renal function. This leads to fluid retention, electrolyte imbalances, and reduced drug clearance, potentially exacerbating clinical outcomes (28). This threshold provides clear guidance for early identification of patients requiring intensive renal function monitoring and fluid management.

The linear relationship between nutritional risk and aspiration pneumonia mortality reflects nutrition’s pivotal role in respiratory rehabilitation and immune function (29). Nutritional status improvement significantly reduces aspiration pneumonia mortality risk regardless of improvement magnitude. This emphasizes the importance of dynamic nutritional assessment and intervention for prognostic enhancement in these patients.

Synergistic interactions indicate nutritional risk and albumin should be jointly evaluated for aspiration pneumonia prognosis. Severe nutritional risk combined with hypoalbuminemia creates high-risk phenotypes through composite effects of immune dysfunction, respiratory muscle weakness, and impaired healing (30, 31). Clinical implications are threefold. First, dual-parameter risk stratification surpasses single-parameter assessment. Second, patients with dual deficits require intensified intervention. Third, even with borderline albumin levels, nutritional optimization remains a viable therapeutic target.

### Strengths

Our study demonstrates how traditional regression and machine learning complement each other in identifying aspiration pneumonia prognostic indicators, rather than producing parallel result sets. This integration is evident in three ways:

First, convergent validation: Both methods independently identified overlapping risk factors (consciousness impairment, age, nutritional status). Regression provided adjusted effect estimates while ML confirmed their importance without parametric assumptions (SHAP analysis, Figures 1I, 2H). This convergence strengthens confidence in these markers’ clinical relevance.

Second, complementary insights: Regression identified *which* factors predict outcomes while ML revealed *how* and *when* these factors operate. For example, regression established albumin as a significant predictor; ML specified the critical 30 g/L threshold below which mortality risk escalates (Figure 2D). Similarly, regression found nutritional risk significant; ML demonstrated its synergistic interaction with albumin levels (Figure 2G), identifying high-risk phenotypes (severe nutritional risk combined with albumin <25 g/L).

Third, integrated roles: Regression guides acute risk stratification for immediate clinical decisions (e.g., consciousness-impaired patients requiring intensive monitoring). ML extends this by identifying clinically actionable risk stratification cues (e.g., albumin <30 g/L; prolonged antibiotic duration as a clinical-course marker) and interaction effects (antibiotic duration × Barthel Index, Figure 1H).

This dual-framework approach leverages regression’s confounder-adjusted association estimates alongside ML’s capacity for revealing nonlinear relationships and interactions, providing a more complete understanding of aspiration pneumonia risk architecture than either method alone.

Our findings illustrate this advantage: traditional analyses identified consciousness-impaired elderly patients requiring intensive monitoring, while machine learning revealed high-risk phenotypes (severe nutritional risk combined with albumin <25 g/L) amenable to modifiable therapeutic strategies. Integration of both approaches—utilizing regression-based predictors for acute risk assessment alongside machine learning-guided long-term prognostic management—holds significant potential for improving aspiration pneumonia patient outcomes.

### Limitations

Despite these strengths, several limitations must be acknowledged. As a single-center retrospective study conducted in Chinese populations, generalizability may be limited when applying findings to other ethnic groups or healthcare systems. Exclusion of immunocompromised patients, active tuberculosis, pulmonary malignancies, and severe hepatorenal dysfunction restricts model applicability in these high-risk populations that may exhibit different clinical patterns. The observational design inherently limits causal inference, demonstrating associations rather than causality, with unmeasured confounders such as social support systems and genetic susceptibility remaining uncontrolled. Several predictors are susceptible to reverse causation. Prolonged antibiotic use, low albumin, and malnutrition likely reflect underlying disease severity rather than causing adverse outcomes. Although our models adjusted for measured confounders, unmeasured factors such as disease severity scores, frailty assessments, social support, and detailed microbiological data remain uncontrolled. Notably, while microbiological culture is fundamental to pneumonia etiology, sputum cultures typically incur a 3–5 day delay and have a notoriously low yield in the advanced-age geriatric population. Consequently, our framework prioritized universally available, immediate clinical parameters to enable rapid (within 24 h) prognostic stratification. Therefore, our findings identify risk markers associated with outcomes but do not establish causal relationships. Prospective studies are needed to determine whether interventions targeting these markers improve outcomes. Additionally, relatively small prognostic sample sizes may limit statistical power for detecting complex interactions, while retrospective characteristics impede standardized assessment protocols for key variables like swallowing function and nutritional status, potentially introducing measurement bias. External validation is required before applying predicted probabilities for clinical decision-making.

### Future perspectives

Future work should externally validate the thresholds and interactions and derive a simplified point-based score for clinical implementation in general inpatient wards and primary care facilities equipped with adequate basic imaging capabilities, to bridge interpretable ML findings with pragmatic clinical decision-making, to bridge interpretable ML findings with pragmatic clinical decision-making. Future prospective, multicenter studies incorporating larger, more diverse populations and standardized assessment protocols are essential for validating these machine learning insights and enhancing clinical applicability.

## Data Availability

The original contributions presented in the study are included in the article/supplementary material, further inquiries can be directed to the corresponding authors.
